# Detailed characterization of the transcriptome of single B cells in mantle cell lymphoma suggesting a potential use for SOX4

**DOI:** 10.1038/s41598-021-98560-1

**Published:** 2021-09-27

**Authors:** Simone Valentin Hansen, Marcus Høy Hansen, Oriane Cédile, Michael Boe Møller, Jacob Haaber, Niels Abildgaard, Charlotte Guldborg Nyvold

**Affiliations:** 1 Hematology-Pathology Research Laboratory, Research Unit for Hematology and Research Unit for Pathology, University of Southern Denmark and Odense University Hospital, Odense, Denmark; 2 OPEN, Odense Patient Data Explorative Network, Odense University Hospital, Odense, Denmark; 3 CITCO, Centre for Cellular Immune Therapy of Hematological Cancer Odense, Odense University Hospital, Odense, Denmark

**Keywords:** Cancer genomics, Haematological cancer, Tumour biomarkers, Tumour heterogeneity, Gene expression analysis, RNA sequencing, Diagnostic markers, Prognostic markers, Cancer genetics, Haematological cancer, Non-hodgkin lymphoma, Molecular medicine, B cells, Transcriptomics

## Abstract

Mantle cell lymphoma (MCL) is a malignancy arising from naive B lymphocytes with common bone marrow (BM) involvement. Although t(11;14) is a primary event in MCL development, the highly diverse molecular etiology and causal genomic events are still being explored. We investigated the transcriptome of CD19^+^ BM cells from eight MCL patients at single-cell level. The transcriptomes revealed marked heterogeneity across patients, while general homogeneity and clonal continuity was observed within the patients with no clear evidence of subclonal involvement. All patients were SOX11^+^CCND1^+^CD20^+^. Despite monotypic surface immunoglobulin (Ig) κ or λ protein expression in MCL, 10.9% of the SOX11 + malignant cells expressed both light chain transcripts. The early lymphocyte transcription factor SOX4 was expressed in a fraction of SOX11 + cells in two patients and co-expressed with the precursor lymphoblastic marker, FAT1, in a blastoid case, suggesting a potential prognostic role. Additionally, SOX4 was found to identify non-malignant SOX11^–^ pro-/pre-B cell populations. Altogether, the observed expression of markers such as SOX4, CD27, IgA and IgG in the SOX11^+^ MCL cells, may suggest that the malignant cells are not fixed in the differentiation state of naïve mature B cells, but instead the patients carry B lymphocytes of different differentiation stages.

## Introduction

Mantle cell lymphoma (MCL) is a subtype of non-Hodgkin’s lymphoma (NHL) with a generally aggressive although heterogeneous disease course^1,2^. One of the primary oncogenic events is the t(11;14)(q13;q32) translocation juxtaposing the cyclin D1 (CCND1) proto-oncogene to the Ig heavy chain (IGH) locus^3^ leading to overexpression of CCND1 and cell cycle deregulation^4^. This translocation is observed in the majority (90%) of MCL cases^1^, but also CCND1 negative cases have been reported, where patients showed overexpression of CCND2^5^ or CCND3^6^. The translocation t(11;14) is presumably acquired in immature pre-B cells of the bone marrow (BM), although the full oncogenic potential develops in mature B cells^2^. The typical immunophenotype is surface expression of CD19, CD20, CD22, CD43, CD79a, CD5 and FMC7 with monoclonal k/λ immunoglobulin (Ig) light chains, while CD23 (also known as FCER2), CD10 (also known as MME), CD200 and BCL6 are typically dim or negative^1,2,7^. In the development of B cells the IGH locus undergoes V(D)J rearrangement forming a unique B cell receptor^8^. As MCL raises from one cell of origin with a unique V(D)J rearrangement, this rearrangement is characteristic for the malignant clone and can be used as a fingerprint for tracking malignant cells^9^.

The development of MCL directs into two major biological and clinical variants; classical nodal MCL and leukemic non-nodal MCL^2,10,11^. Classical MCL has usually an aggressive clinical course and typically involves lymph nodes and other extra-nodal sites at presentation. This form presents with a higher degree of genomic instability^2,10,12^, and is positive for SOX11, an acknowledged specific marker of MCL^13,14^. This subtype originates in a B cell that is unexposed to the germinal center and therefore has no or low percentage of IGHV somatic hypermutations and an epigenetic methylation signature, corresponding to naive B cells^2^. The acquisition of additional molecular aberrations can lead to more aggressive variants^2,10,12^. Leukemic non-nodal MCL is negative for SOX11 and typically involves peripheral blood (PB), BM, and spleen^2,10,15,16^. This subtype originates in a B cell that has been exposed to the germinal center and therefore has hypermutated IGHV and a methylation signature corresponding to memory B cells^2^. These cases are often clinically indolent with superior outcome compared to classical MCL, but may evolve to aggressive disease when additional aberrations occur^2,10,15,16^. Classical MCL is the most common type, while leukemic non-nodal MCL represents only 10–20% of patients^16^. Histological variants include classic MCL with monomorphic lymphoid proliferation of small to medium sized cells, where the proliferative activity usually is low^2^. More aggressive types include the blastoid and the pleomorphic variants^2^, which constitute 10%^17^ to > 20% of all MCLs^18^, respectively.

SOX11 is a member of the SOXC protein family, which also includes SOX4 and SOX12^19^. The three SOXC proteins exhibit overlapping expression patterns and molecular properties, and may act in redundancy to control developmental, physiological and pathological processes^19–22^. SOX11 is a transcription factor that has been reported to promote angiogenesis^23^, migration and adhesion of MCL cells to stromal cells^24^, thereby promoting cell-adhesion-mediated drug resistance^24^. It can impact MCL cells by augmentation of BCR signaling^25^, suppression of BCL6^26^ to avoid MCL cells entering the germinal center thereby keeping IGHV unmutated, and by activation of PAX-5 thereby blocking the maturation to plasma cells^27^. SOX4 is a homologous transcription factor^19,22^ required for development and differentiation of lymphocytes^28–30^ and was found to be expressed in pro-B cells^31^. In acute myeloid leukemia, SOX4 was shown to be an important factor in leukemogenesis^32,33^, and high expression of SOX4 was a poor prognostic factor^32^. In pre-B acute lymphoblastic leukemia, SOX4 was found to be required for survival, progression and proliferation^34,35^ and correlates with poor clinical outcome^34,35^. Elevated SOX4 expression has also been found in a wide variety of solid cancers, where mostly oncogenic roles have been reported^22,36^.

Collectively, MCL is considered a highly heterogeneous disease with respect to clinical presentation and prognosis^37,38^, and high molecular variation with subclonal intra-tumor heterogeneity has been demonstrated already at diagnosis^39–41^. Presence of multiple subclones at diagnosis has been associated with decreased relapse-free survival, suggesting a prognostic impact^42,43^. The molecular heterogeneity of MCL makes it challenging to define standard therapies^12^ and is a plausible explanation for the diverse outcomes of this B malignancy. In this study, we investigated the transcriptome of MCL cells from eight diagnostic BM samples to provide insight into the complex and diverse molecular architecture of MCL at the single-cell transcriptomic level in the perspective of commonly used molecular pathology markers.

## Results

Single cell RNA-sequencing (scRNA-seq) was performed on CD19^+^ B lymphocytes isolated from diagnostic bone marrow aspirates (Fig. S1 and Table S1) of eight patients diagnosed with MCL. A total of 30,565 cells were collected using the Chromium platform. On average, 3800 cells from each patient passed the quality threshold and were included in the downstream analyses (1018–6668 cells, Table S2). In general, patient samples 2, 4, and 6 displayed superior quality relative to the rest of the cohort, with a median of 772–1151 expressed genes per cell versus 309–517 (Table S2). Of note, the quality of the sequencing output was in concordance with higher clonal infiltration of bone marrow, cell purity and RNA integrity (Table S1–S3).

### Global transcriptomic profiles of MCL bone marrow B lymphocytes

**Figure 1:**
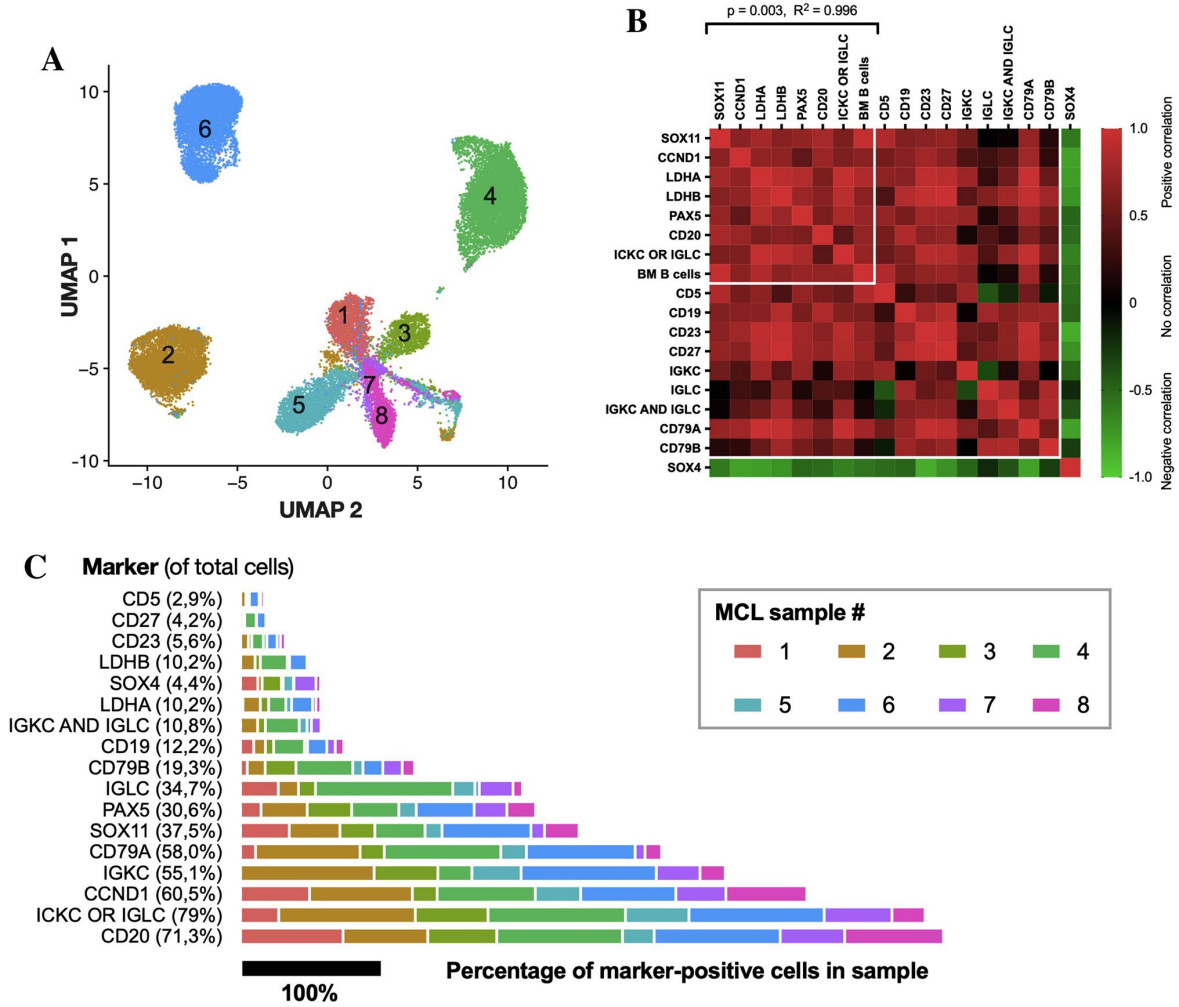
Clustering of combined single-cell transcriptomes, expression and correlation of molecular pathology MCL markers. The MCL cohort displayed heterogeneous expression profiles, forming distinct clusters of single-cell transcriptomes, except for co-located sample 7 and 8. Cells are colored and numbered according to patient origin (**A**). A highly significant positive correlation (red) was found between the percentage of SOX11expressing cells and cells expressing frequently used molecular pathology markers in MCL (**B**) including CCND1, LDHA/B, PAX5, CD20, immunoglobulin κ/λ light chain (IGKC/IGLC) (*p* = 0.003). The fraction of SOX11 positive cells also showed a positive correlation with the percentage of B cells in the bone marrow samples known from the clinical flow cytometry analysis (*BM B cells*, *p* = 0.003). The rest of the listed markers showed strong multicollinearity and were hence excluded in the linear regression. The percentage of SOX4^+^ cells was negatively correlated (green), with other markers and associated with residual non-malignant pro-/pre-B cells (see also Fig. 2b). The percentage of cells expressing molecular pathology markers were calculated for the individual patients (bars) and from the total single cell cohort (numbers) as described in the methods section (**C**). The total single cell population (numbers) and the cells from individual patients (bars) were markedly positive for CCND1, CD20 and κ/λ (IGKC/IGLC), while merely 37.5% of the total sequenced cells were SOX11^+^, varying from 9.8 to 64.6%. 10.8% of the combined cohort was found to harbor dual expression of κ and λ light chains.

Joint dimensional reduction, using UMAP (Uniform Manifold Approximation and Projection^44^) of the single cell transcriptomes to low dimensional feature space showed a resolution to discern discrete transcriptomic populations of the individual cases (Fig. 1A). This transcriptomic heterogeneity was in concordance with the general notion of inter-patient heterogeneity of MCL. A significant correlation was found between SOX11 expression (*p* = 0.003, R^2^ = 0.996, Fig. 1B) and molecular pathology markers frequently applied in diagnosis of MCL, whereas SOX4 negatively correlated with these markers.

### Expression of molecular pathology markers frequently applied in diagnosis of MCL at single cell transcriptomic level

**Figure 2:**
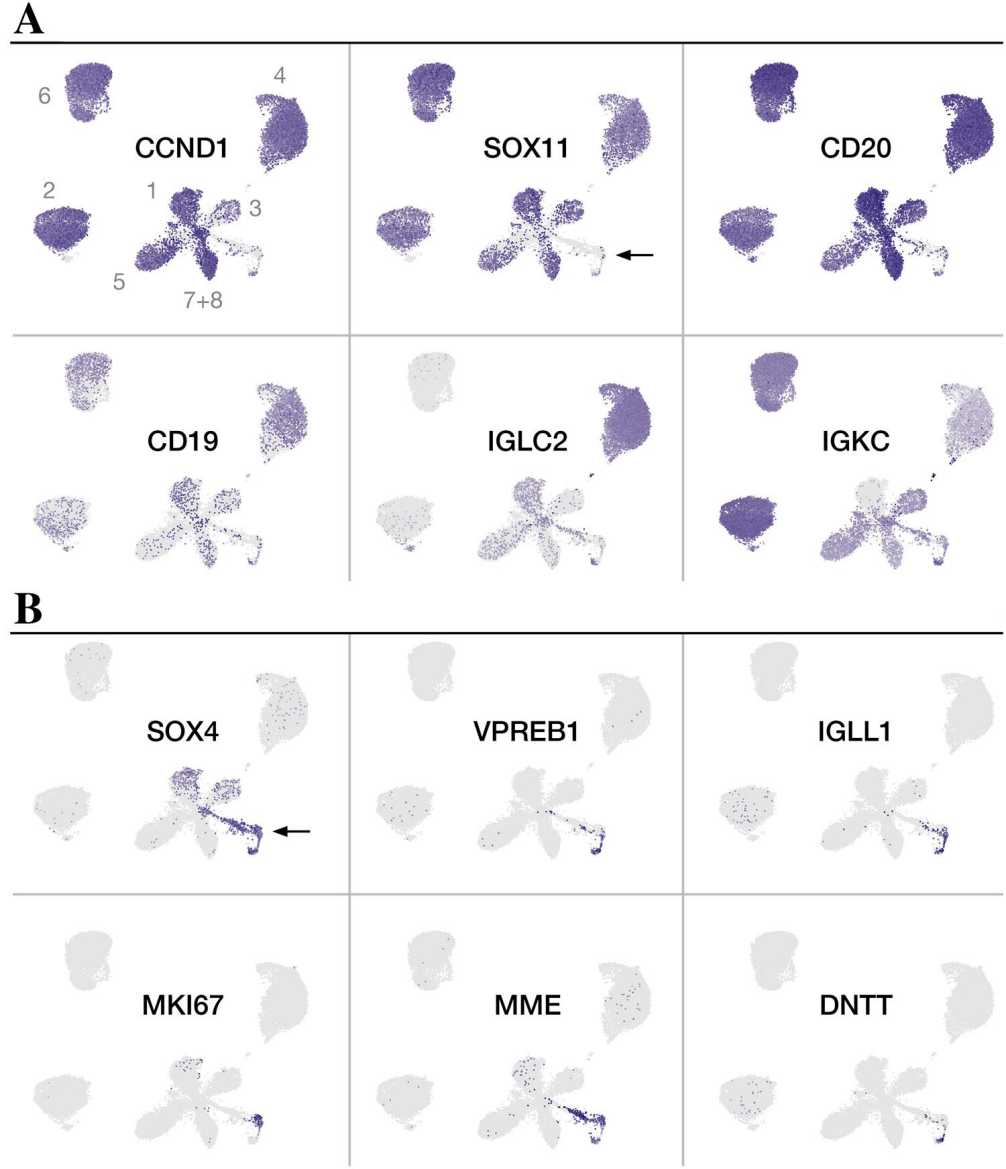
Global expression of molecular pathology markers frequently applied in diagnosis of mantle cell lymphoma (**A**) and markers associated with immature B cells (**B**) in combined mantle cell lymphoma (MCL) single cell transcriptomes. The resolved representations of cells from each MCL patient (pt.) were in general agreement with molecular pathology markers of MCL (**A**), although CD19 was only positive in a fraction of the single cells at the transcriptional level (12.2%). The dominating Ig κ and λ light chain restriction could be transcriptionally identified, with pt. 2, 3, 5, 6, 7, 8 being κ, and 1 and 4 being λ, the average double-positive was 7%, ranging from 0 (pt. 1) to 24% (pt. 4). CCND1 and CD20 were the most widely expressed molecular pathology markers. Markers of immature pre-pro B cells and pro-B cells were expressed in SOX11^-^ areas containing cells from multiple patients (**B**). Three patients (pt. 1, 3 and 7) had a substantial cell fraction expressing SOX4 co-localized with SOX11^+^. Purple indicates positive expression, and the intensity of the color reflects increased expression. MME: also known as CD10.

Concordant with the clinical laboratory results, all patients were positive for SOX11 and CCND1, while only 37.5% of the total single-cell population expressed SOX11 at a detectable level (ranging from 9.8 to 64.6% in the individual patients, Figs. 1C, 2, Table S4–S5), and 71.1% (range: 26.4–81.4%) of the SOX11^+^ cells co-expressed CCND1 (Table S5). Looking into the two other homologous SOXC family members, 4.4% of all cells expressed SOX4 (range: 0.4–16.0%), while being negative for SOX12 (Figs. 1C, 2, Table S4–S5). Three patients (1, 3 and 7) harbored a substantial SOX4 positive fraction within the SOX11 expressing cells of 19.7%, 13.3% and 12.2%, respectively (Figs. 1C, 2, Table S4–S5).

Generally, the combined population was positive for CD20 and did not express the transcripts for CD5, CD19, CD23 or CD27 (Figs. 1C, 2, Table S4–S5), although the profiles varied patient-wise. Patient 5 was almost completely devoid of measurable CD5 and CD19 transcripts in the SOX11^+^ population (Table S5). 10.8% of all cells (range: 0.2–24.5%), and 10.9% of SOX11^+^ cells (range: 0.3–25.8%), were found positive for both κ and λ Ig light chain genes (Figs. 1C, 2, Table S4–S5). While 90.5% of all SOX11^+^ cells were positive for IgM (range: 51.6–98.7%, Table S5), only 17.8% expressed IgD (range: 0.8–43.1%, Table S5). All patients harbored SOX11^+^ cells expressing IgA (range: 6.1–35.1%, Table S5) and IgG (range: 0.8–14.6%, Table S5), and a small SOX11^+^CD27^+^ fraction (3–9.6%, Table S5) was detected in patient 2, 4 and 6. Additionally, minor compartments of SOX11^+^CD23^+^ cells were detected in all patients (range: 1.4–9.2%, Table S5).

Differential expression analysis with gene set enrichment analysis (GSEA, data not shown) identified non-malignant pro-/pre-B cells within the cohort significantly different from the malignant and SOX11^+^ cells. These cells were enriched in bone marrow pre-B markers (GSEA, marrow CD34^+^ pre-B^45^, *p* = 6.9 * 10^–63^, q_FDR_ = 4.81 * 10^–59^, 40/98 gene overlap) and markers of lymphocyte progenitors (GSEA^46^, *p* = 3.33 * 10^–43^, q_FDR_ = 1.16 * 10^–39^, 41/289 gene overlap) such as SOX4, IGLL1 (also known as IGL5/CD179B/VPREB2), DNTT, VPREB1, and CD10 (Fig. 2B). As expected, the non-malignant B cells were co-localized within the cohort by transcriptional clustering and did not show evidence of Ig light chain restriction (Figs. 1A, 2).

### Local transcriptomic profiles of malignant cells

Next, we explored how expression profiles varied among purified CD19^+^ bone marrow cells within the individual patients. The most frequent significantly altered genes from unsupervised clustering (Seurat cluster resolution 0.2–0.4, data not shown) were related to NFκB signaling (14 genes), apoptosis (9 genes), IL2/STAT5 (5 genes) and TP53 pathways (5 genes) (GSEA, hallmark gene sets, 5.39 * 10^–5^ > *p* > 4.7 * 10^–18^, 3.85 * 10^–4^ > q_FDR_ > 2.35 * 10^–16^).

**Figure 3:**
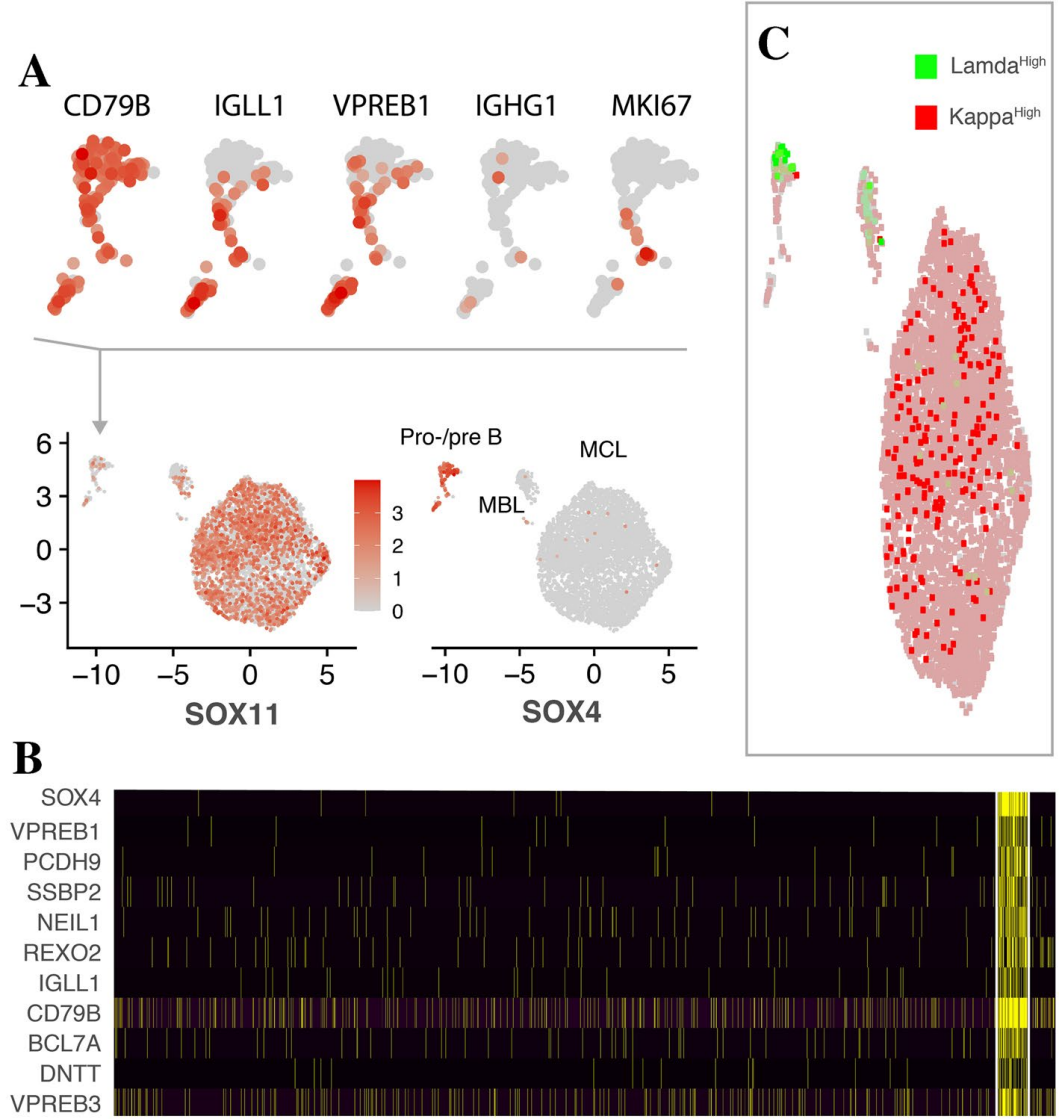
Cluster trio of patient 2. The resolved subpopulations could be identified as a large cohort of lymphoma cells (MCL), a minor subpopulation assumed to reflect monoclonal B cell lymphocytosis (MBL), and a relatively small cluster of cells expressing (red) IGLL1 with a possible role in pro-B cell to pre-B cell differentiation, with little evidence of isotype-switched B cells (Pro/pre B) (**A**). The profile of this cluster was significant for immature B cells of either pro- or pre-B cells (yellow indicates positive expression) (**B**). Importantly, immature SOX11^+^ cells was discernible. The minor population assumed to represent an MBL clone, resolved from flow cytometry, was positive for both immunoglobulin light chain λ (green) and κ (red) genes (IGLC, IGKC) as was a fraction of immature cells (**C**), while the MCL clone was λ negative. Also, the MBL cluster was enriched in CD23 and markers of isotype-switched B cells (IgA and IgG) (*not shown*).

Algorithmically defined clusters (shared nearest neighbor (SNN) clustering) of each patient did not provide any clear evidence of multiple clones or subclones within the malignant population, with the exception of Patient 2 (Fig. 3). The general lack of multiple clones and subclones was supported by subsequent deep sequencing of immunoglobulin heavy chain gene rearrangements (data not shown) using the LymphoTrack assay [704,889 mapped IgH reads (537,282–858,000)]. Except for patient 2, all eight patients were found to have a single malignant B cell clone since only one V(D)J rearrangement was detected by deep sequencing of immunoglobulin heavy chain gene rearrangements (data not shown). In patient 2, two different rearrangements with different J genes was found. Although the minor clone only constituted ~ $\hspace{0.17em}1$%, this was indicative of two different B cell clones in this patient, and may be in consistence with this patient having a small monoclonal B cell lymphocytosis (MBL) clone according to the clinical flow data (Table 1). Subclone analysis based on somatic hypermutation showed no clear evidence of subclonal evolution in any of the samples.

**Table 1:** Clinical patient information.

Patient	1	2^a^	3	4	5	6	7	8
Sex	Female	Female	Male	Male	Male	Female	Male	Male
Age	86	72	78	62	67	72	68	88
Date of diagnosis	July 2017	April 2018	May 2017	Nov 2017	Nov 2017	July 2017	May 2019	Oct 2019
Infiltration of MCL in bone marrow (% of vital cells)	28%	38.2%	11.6% (based on previous sample)	32% (based on previous sample)	4.3%	71.2%	4.6%	6.08%
Fraction of MCL cells from CD19^+^ cells in bone marrow	100%	93.6%	88.3%	95.5%	100%	100%	82.8%	57.5%
Nodal involvement	Yes	Yes	Yes	Yes	Yes	Yes	Yes	Yes
Splenomegaly	No	No	Yes	Yes	No	No	No	No
Extra-nodal sites	Lungs	Coecum	No	No	No	Pleural fluid	Tongue	No
Leukocyte count in blood	17.4 * 10^9^/L	16 * 10^9^/L	12 * 10^9^/L	–	–	18.4 * 10^9^/L	6.17 * 10^9^/L	8.48 * 10^9^/L
Morphology	Blastoid	Classical	–	Classical	Pleomorphic	Classical	Classical	Pleomorphic
Cyclin D1^+^	Yes	Yes	Yes	Yes	Yes	Yes	Yes	Yes
SOX11^+^	Yes	Yes	40%	Yes	Yes	Yes	Variable	Yes
Immuno-phenotype	CD19^+^ CD20^+^ CD22^+^ λ^+high^ CD5^+^ CD79b^+^ CD10^partly^	CD19^+^ CD20^+^ CD22^dim^ κ^+^ CD5^+^	CD19^+^ CD20^+^ κ^high^ CD79b^+^ CD23^partly^ CD22^+^	CD19^+^ CD20^+^ CD22^+^ λ^+^ CD5^dim^ CD79b^+^ CD27^dim^	CD19^+^ CD20^+^ CD22^+^ κ^+^	CD45^+^ CD19^+^ CD20^+^ CD22^+dim^ κ^high^ CD79b^+^	CD19^+^ CD20^+^ CD5^+^ κ^+^	CD19^+^ CD20^+^ CD5^+^ κ^+^ CD38^+^
MIPIc score at diagnosis	8.5	7.1	7.2	6.3	8.4	7.3	5.8	7.8#
Current status	Dead from MCL	Alive	Dead (not from MCL)	Alive	Alive	Alive	Alive	Alive
Time to progression	1st relapse: 10 months No response to 2nd line therapy	Not reached	Not reached	Not reached	Not reached	Not reached	Not reached	Not reached
Follow-up time	15 months	36 months	47 months	41 months	41 months	45 months	23 months	18 months

Patient information was obtained from the clinical records. MCL infiltration was determined by flow cytometry analysis of bone marrow (BM) cells and calculated as percentage of the vital cells in the sample. The fraction of MCL cells from CD19^+^ cells was calculated, based on the flow cytometry clinical laboratory data, by dividing the percentage of all CD19^+^ cells including MCL cells, non-malignant B cells and plasma cells ($\sim$ 70% of plasma cells weakly express CD19) by the percentage of monoclonal CD19^+^ cells in the BM sample. In all patients, the MCL cells were positive for CD19 and CD20 and showed immunoglobulin light chain restriction. All patients had nodal involvement, bone marrow involvement, and were positive for both cyclin D1 and SOX11 as evaluated by immunohistochemistry staining of lymphocytes in lymph node and bone marrow biopsies. Apart from this, the patients were, in consistence with the pathology of MCL, heterogeneous in their clinical presentation.

^a^Patient 2 had a small (2%) monoclonal B cell lymphocytosis (MBL) clone with a chronic lymphocytic leukaemia (CLL)-like profile CD19^+^CD22^+^CD20^dim^CD5^+^lambda^dim^. #LDH was unsure.

The two distinct subclusters of Patient 2 (Fig. 3A) were identified as one expressing markers of immature B cells (pro/pre-B cells, Fig. 3B), and the other suggestive of MBL with a λ positive CLL-like profile in line with the clinical flow cytometry data from this patient (Table 1). This cluster, constituting $\sim$ 3.3% of the cells, was significantly increased for Ig light chain λ genes (Fig. 3C, IGLC1, IGLC2, 3.6–12 × fold-change, 58.1–75% positive cells in this cluster versus 2.4–8.8% in other clusters), CD23 (2.7 × fold-change, 42% positive cells in this cluster vs 5% in other clusters) and isotype-switched B markers (IgG, IgA) along with MEF2C, FCRL1 and other B cell markers. Although the generated clusters were strongly indicative of pro/pre-B cells and MBL, respectively, both contained a small and partly SOX11 positive cell subset (13%, 2.5 × expressional decrease).

**Figure 4:**
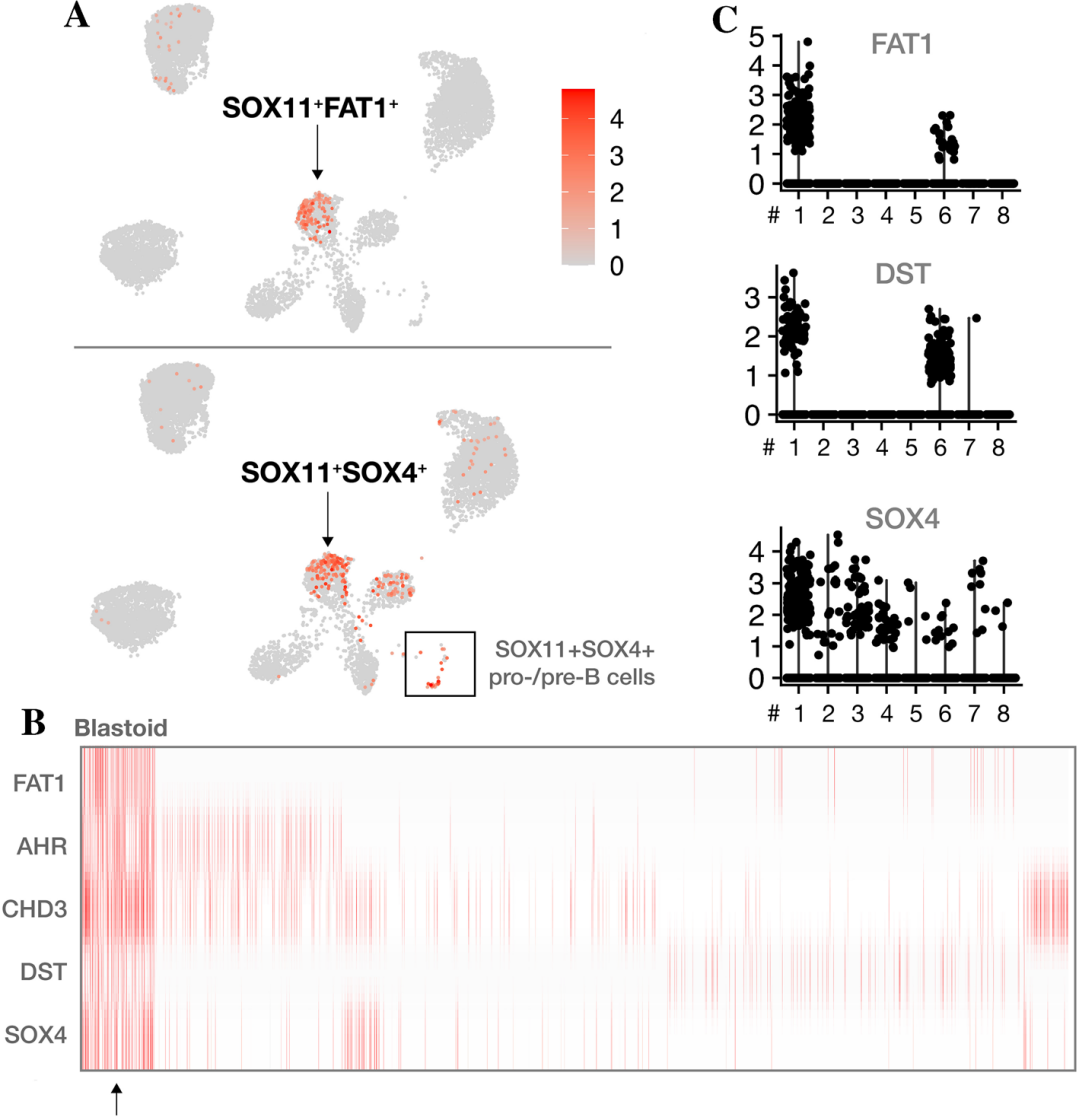
Expression signature of the blastoid mantle cell lymphoma case. Patient 1 had highly specific FAT1 expression (red, upper) among SOX11^+^ cells, while SOX4 expression (red, lower) was also found in pt. 3, 4, and 7 as well as in a small fraction of pro-/pre-B cells (**A**). A large fraction of these cells was also significant for an AHR^+^, CHD3^+^, DST^+^, SOX4^+^ expression (red) signature (**B**), providing potential evidence of a more immature cell type based on previous findings in lymphoblastic leukemia/lymphoma. While the blastoid patient had a very high fraction of malignant SOX4^+^ cells, comprising 19.9% of the SOX11^+^ population and no evidence of healthy pro-/pre-B cells, it was not specific for SOX4^+^ cells in general. Violin plots show the expression (y-axis) of genes in each patient (x-axis) (**C**).

Apart from the results related to Patient 2, one of the most significant findings from the entire cohort of malignant SOX11^+^ cells was the identification of distinct markers from blastoid MCL cells of Patient 1, e.g. protocadherin FAT1 expressing cells (Fig. 4). FAT1, almost exclusively located in the bone marrow B lymphocytes of patient 1, was expressed in a compartment of SOX4, Aryl Hydrocarbon Receptor (AHR), Chromodomain Helicase DNA Binding Protein 3 (CHD3) and Dystonin (DST) positive cells (Fig. 4B). The blastoid case was evidently monoclonal, λ chain restricted, with a very small but identifiable number of malignant MKI67 expressing cells (data not shown). We did not observe any informative individual features in the rest of the cohort.

## Discussion

The complex and diverse molecular architecture of MCL is a plausible explanation for the diverse outcome of the disease. However, it is still unclear what cellular architecture is comprised within the patients. To gain insight into this heterogeneity at single cell level, we performed single cell mRNA sequencing of the purified CD19^+^ fraction of diagnostic bone marrow aspirates from eight MCL patients. The inter-tumor heterogeneity was striking as previously reported in MCL^47^. However, in contrast to the subclonal involvement, as shown on unsorted mononuclear cells by two recent scRNA-seq studies^47,48^, the transcriptional profiles observed in this study were rather unremarkable with a homogeneous continuum of expression patterns observed for the malignant cells.

CD19 and CD5 expressions were not detected in all cells at the transcriptional level, as reported previously^48^, indicating a relatively low mRNA abundance or a poor correlation of the proteins and mRNA. Neither CCND1 nor SOX11 was expressed in all malignant B cells from MCL patients and not all SOX11^+^ cells expressed CCND1, as observed previously^47^. Collectively, this phenomenon may be explained by transcriptional bursting^49–51^ or simply that the expression levels of the genes were below the detection limit or resolution of the scRNA-seq assay.

We noted that the commonly used markers, expression of κ and λ Ig light chains, were found to be suboptimal for clonal identification at single cell mRNA level, since co-expression of the transcripts was detected in 10.9% of the SOX11^+^ single cell population, although largely concordant with the light chain restriction observed in the clinical laboratory analyses. The limitations in the number of recorded cells, the panel design (optimal for diagnosis but not for κ and λ co-expression), and difference in cell preparation used for the diagnosis staining did not enable us to confirm the κ/λ protein co-expression in the patient clinical flow cytometry data. Previous studies have reported that dual protein expression of κ and λ Ig light chains could be demonstrated in B cell malignancies^52,53^ and in healthy B cells^54,55^. These observations suggest that this phenomenon is not rare^53^, at least in MCL, and should be considered accordingly, when assessing the clonal burden by means of κ/λ transcript ratios. It may also suggest that some MCL cells further rearrange Ig light chain genes, or that some of the MCL cells may originate from immature B cells with dual expression^52^.

Not surprisingly, the expression levels of the classical molecular pathology markers used in MCL diagnostics, e.g. SOX11, CCND1, PAX5, CD79B and CD20 were correlated. Although the percentage of measurable CD19 and CD5 positive cells was low, the fractions showed positive correlation with the other markers, whereas a negative correlation was observed between SOX4 and SOX11 in the combined BM B lymphocyte population. This was ascribed to the presence of healthy pro-/pre-B cells, in spite of three patients (patient 1, 3 and 7) co-expressing the transcripts of both of the SOXC proteins. Unexpectedly, the memory B cell marker CD27 and the CLL marker CD23 positively correlated with diagnostic MCL markers. We observed that CD23, frequently used to differentiate CLL from MCL, was present in a subset of the SOX11^+^ MCL cells, supporting previous findings that some MCLs are CD23^+^^56,57^. The majority of cells were CD20^+^CD27^−^ indicating that few or no memory B cells were present. In the same line, most cells expressed IgM, indicating mainly naïve mature B cells, concordant with that of CD19^+^ bone marrow cells and MCL cells of the nodal type.

All patients had SOX11^+^ cells expressing transcripts of isotype-switched IgH in addition to a small CD27^+^SOX11^+^ fraction observed in patient 2, 4 and 6 suggesting that some MCL cells may potentially be antigen-experienced, although expected to originate from naïve B cells. In line with this observation, MCL cells expressing CD27 protein, and transcripts for IgA and have been previously reported^58–61^, in addition to sporadic accounts of IgA^60^ and IgG surface protein expression^61^. In CLL, resembling MCL in several ways, cells expressing IgG and IgA transcripts with a V(D)J rearrangement identical to that of the IgM+ clone were observed but these cells only expressed IgM protein^62^. Our data thus add to the current knowledge by showing that such transcript profile is found in a specific cell fraction and support a role for antigen involvement in MCL, as previous suggested^59,61,63^.

A subset of cells in patient 1, 3 and 7 was found to express the immature pro-B cell marker, SOX4, suggesting that not all MCL cells originate from mature, naive B cells and maybe some patients carry a reservoir of more immature malignant cells, which would support the hypothesis of multiple cellular origins of MCL^61^. Additionally, it suggests a potential clinical role for SOX4 to supplement one of the most important clinical MCL markers, and transcription factor homologue, SOX11. It is known that SOX4 is required for the development and differentiation of early B cells^31^. We observed that in MCL BM, the non-malignant pro-/pre-B cells were characterized by SOX4 expression, whereas the clinically defined blastoid MCL case (patient 1) was marked by a subset of cells expressing both SOX11 and SOX4 together with FAT1. Although further studies are required to establish its role in blastoid MCL, the latter was found to be exclusively expressed in this particular patient (20% of SOX11^+^ cells). FAT1 has been described as having both tumor suppressive^64–68^ and oncogenic^69–72^ roles, depending on the context. In the context of MCL, somatic mutations in the FAT1 gene have been reported in a few patients^41^, but its role in MCL has, to our knowledge, not yet been described. Interestingly, evidence points to FAT1 being a specific marker in acute lymphoblastic leukemia (ALL)^70,73^. Additionally, the blastoid case presented here, also expressed the pre-B-ALL marker CD10 in a subset of SOX11^+^ cells. It is known that SOX4 plays a central role in the survival of malignant lymphoblasts^34,35,74^ and possibly predicts clinical outcome^34^. In cervical squamous cell carcinoma, FAT1 positively correlated with SOX4, and upregulated it to promote migration and invasion of cancer cells^72^. High FAT1 levels also predicted poor survival^72^. Thus, these markers, posed for further investigation, may help to establish the differentiation state and possibly prognosis of MCL. This raises the question of a possible prognostic value for the fraction of non-malignant SOX4^+^ or immature SOX11^+^ cells.

The clinical marker KI67, which is often employed for the prognostication of MCL, was restricted to a compartment of SOX4^+^ pro-/pre-B cells. Since CD19^+^ BM cells were sorted as singlets, cell doublets, probably including proliferating cells, were excluded therefore supporting the few number of KI67 positive MCL cells in the single cell data. Only in the blastoid case, a very small number of KI67 expressing malignant cells was found, which could be due to a high KI-67 staining index observed by immunohistochemistry for this blastoid MCL patient.

The samples with the highest quality (patient 2, 4, and 6) reflected the highest degree of MCL infiltration in the bone marrow samples and the highest spatial resolution. The transcriptional profile at the single-cell level is known to be noisier than bulk analyses^75^. This may partially be attributed to technical dropout in reverse transcription, extensive amplification of the small amount of RNA or may be caused by biological mechanisms, such as cell cycle or transcriptional bursting^49,76^. For this reason, the reported findings are preliminary and hypothesis-generating only, and must be further explored and confirmed.

In conclusion, our study confirms the inter-patient heterogeneity of MCL and provides insight into molecular pathology markers analyzed in MCL diagnostics at the single-cell transcription level. Importantly, the coinciding FAT1 and SOX4 mRNA expression in the SOX11^+^ cluster of malignant cells was specific for the blastoid case and may directly hold evidence of cells with a more immature profile and not just reflect a distinct morphology. Thus, it may be an important functional gene expression signature in this morphological subtype of MCL. We showed that SOX11 expression positively correlated with the mRNA expression of molecular pathology markers frequently applied in MCL diagnostics. Importantly, we identified a fraction of MCL cells expressing transcripts associated with antigen-experienced B cells in addition to CD23 positive cells, otherwise differentially associated with CLL, and co-expression of κ and λ Ig light chain genes.

## Materials and methods

Mononuclear cells (MNCs) from 8 patients (62–88 years, Table 1) diagnosed with MCL at Odense University Hospital (OUH), Denmark, from 2017 to 2020, were isolated by Ficoll (GE Health Care, Chicago IL, USA) gradient centrifugation from bone marrow (BM) at diagnosis and either stored in RPMI medium (Gibco, Thermo Fisher Scientific, Waltham, MA, USA) with 20% FBS (Gibco, Invitrogen, Thermo Fisher Scientific, Waltham, MA, USA) and 10% DMSO (Sigma-Aldrich, St. Louis, MI, USA) in liquid nitrogen for subsequent cell isolation, or in mRNA lysis buffer (Roche, Basel, Switzerland) and stored at − 80 °C. All patients had nodal involvement, BM involvement, and were positive for both cyclin D1 and SOX11, determined by immunohistochemistry with an otherwise heterogeneous clinical presentation.

### Single cell sample preparation and cell sorting

2.76–10 million cells from cryopreserved MNCs were stained with conjugated antibodies for CD19 (clone HIB19, BD Bioscience, Franklin Lakes, NJ, USA) and CD3 (clone SK7, BD Bioscience, Franklin Lakes, NJ, USA) in Hank’s Balanced Salt Solution (HBSS; Gibco, Invitrogen, Thermofisher Scientific, Waltham MA, USA) 2% FBS (Gibco, Invitrogen, Thermofisher Scientific, Waltham MA, USA) after blocking with Fc Receptor Block (BD Bioscence, Franklin Lakes, NJ, USA). Subsequently, cells were stained with Annexin V (Biolegend, San Diego, CA, USA) and 7AAD (BD Pharmingen, BD Bioscience, Franklin Lakes, NJ, USA) in Annexin V binding buffer (Biolegend, San Diego, CA, USA). The 7AAD^-^Annexin V^-^CD3^-^CD19^+^ B cells were sorted (Fig. S1) on a FACS ARIA III (BD) using a 100 μm nozzle and attained a purity of 75.6–99.7% from singlet gate and 12.3–62.8% from total (Suppl. Table S1, Fig. S2), indicating a higher fraction of apoptotic cells and debris in some samples.

When sufficient number of cells were available (> 15,000 events, 4/8 samples), viability was assessed with trypan blue (Sigma-Aldrich, St. Louis, Mi, USA) staining showing that the median percentage of viable cells was 92.9% (range 91.3–100%). The sorted 7AAD^-^Annexin V^-^CD3^-^CD19^+^ B cells were fixated according to 10 × Genomics protocol (Suppl. methods) and stored at − 80 °C prior to sequencing. Fixated cells were rehydrated prior to single cell RNA sequencing according to the protocol from 10X Genomics (Suppl. methods). RNA integrity number (RIN) was assessed using Bioanalyzer RNA 6000 Pico Kit (Agilent Technologies, CA, USA) on an Agilent Bioanalyzer 2100, reaching RIN of 8.3–9.1 for patient 2–4 and 6, while unavailable for patient 1, 5, 7 and 8 (Table S2).

### Single cell RNA library preparation and sequencing

Cellular suspensions were aimed at 10,000 cells per sample loaded onto a Chromium Next GEM Chip G together with Next GEM Single Cell 3’ v3.1 Gel Beads (10 × Genomics, Pleasanton, CA, USA) and partitioning oil to generate single cell Gel Beads-in-Emulsion (GEMs), followed by reverse transcription at 53 °C. GEMs were broken using Recovery Agent (10 × Genomics), and the resulting cDNA was cleaned up with DynaBeads MyOne Silane Beads (Thermo Fisher Scientific, Waltham, MA, USA) and amplified by PCR using Single Cell 3′ GEM Kit v3.1 with subsequent cDNA clean-up (SPRIselect Reagent Beads, Beckman Coulter, Brea, CA, USA). Concentrations were measured with Qubit dsDNA HS Assay Kit (Thermo Fisher Scientific, Waltham, MA, USA). Enzymatic fragmentation, end-repair, and A-tailing were performed in one step using Single Cell 3′ Library Kit v3.1, and were followed by a double-sided size selection using SPRIselect Reagent Beads. After a final double-sided size selection, the fragment sizes and concentrations were measured using QIAxcel DNA High Resolution Kit (1200) (Qiagen, Hilden, Germany) and KAPA Library Quantification Kit (Roche, Basel, Switzerland), respectively. Finally, the single-cell RNA (scRNA) libraries were sequenced on a NovaSeq 6000 (S1 Reagent Kits, Illumina, San Diego, CA, USA) platform, aiming at 40,000–60,000 reads per cell. Sequencing output per flow cell (2 × 50 bp) were 259 (sample 1–4) and 224 Gb (sample 5–8) with > 90% of the base calls reaching a quality score of 30 or more.

### Processing and analysis of single cell RNA sequencing data

Sequencing raw data demultiplexing was performed with Cell Ranger *mkfastq* (Cell Ranger v3.1.0, 10x), and subsequent alignment to reference genome GRCh38 (prebuilt, 10x, GENCODE v32/Ensembl 98) was performed with STAR^77^ through Cell Ranger *count*. Merging of data from all patients and cross-sample normalization, as well as intra-/inter-sample differential expression analyses were performed in R (R 3.6, Seurat 3.2^78^). Doublets, low quality cells and empty droplets were removed based on feature counts, mitochondrial read fraction and expression of B markers. The thresholds for the filtering were defined by Tukey's fences (± 1.5 IQR) and outliers were removed from further analysis. Cells were transcriptionally restricted to positive expression of at least one of the following B-cell markers: IgH genes, Ig light chain genes, CD20, CD19 or CD79A/B. We defined positive expression of a given gene as more than 0.01% percent of counts originating from the specific *feature*, using Seurat function *PercentageFeatureSet* with regex pattern “^*feature*$”^78,79^. Multiple regression of molecular pathology markers frequently analyzed in diagnosis of MCL was performed in R, using the linear model (lm).

We combined the single cell transcriptomes of CD19^+^ B cells from all eight patients and jointly visualized these using Uniform Manifold Approximation and Projection (UMAP, Fig. 1A) for dimensional reduction of gene expression profiles to low-dimension feature space. Clustering of cells was performed with SNN clustering using cluster resolution 1.5 for the merged analysis and ranging from cluster resolution 0.2–0.5 for analysis of individual samples, selected according to overall quality. Clusters of MCL cells were distinguished from non-malignant B cells based on gene expression profiling (GSEA), monoclonality (restricted light chain expression) and expression of SOX11.

A total of 30,565 cells were sequenced (1018–6040 per sample) with mean reads per cell above 82,511 for 6 out of 8 samples (range 82,511–254,028 reads), while being lower, 25,422 and 33,591 mean reads per cell, for two samples (sample 5 and 8). The median unique molecular identifier counts per cell were 434–2704, while the median genes per cell was 309–1151 (Table S3). For samples 3, 5, and 7, the median genes per cell was less than 500 genes (309–460).

### Sequencing of clonal rearrangements

DNA from MNCs was extracted using the MagNA LC DNA isolation kit (Roche), and quantification of DNA performed using the Qubit 2.0 dsDNA HS assay kit and a Qubit 2.0 fluorometer (Thermo Fisher Scientific). A minimum of 50 ng DNA (50–78 ng) was used for next generation sequencing (NGS) of the immunoglobulin heavy chain clonal rearrangement using the LymphoTrack Dx IGH FR1 assay (Invivoscribe, San Diego, CA, USA) and a Prime Ion Gene Studio S5 sequencer (Ion Torrent; Thermo Fisher Scientific) according to the provided instructions. Data were analysed using the LymphoTrack Dx Software S5 package (Invivoscribe, San Diego, CA, USA). Each merged clonal sequence was evaluated for evidence of somatic hypermutation (SHM), as described by the supplier (Invivoscribe).

### Ethical considerations

Informed consent was obtained from all patients. The project was approved by the National Committee on Health Research Ethics, Denmark (Approval No. 1605184), and data were handled in accordance with the requirements of the Danish Data Protection Authority.

## Supplementary Information

Supplementary Information.

## Data Availability

Sequencing data (10 × Cell Ranger output) is available at https://doi.org/10.6084/m9.figshare.14743233. Please cite paper accordingly.
