# Supplementary material for: Detailed characterization of the transcriptome of single B cells in mantle cell lymphoma suggesting a potential use for SOX4
Source: Sci Rep. 2021 Sep 27;11:19092. doi: 10.1038/s41598-021-98560-1 (PMC8476518; doi:10.1038/s41598-021-98560-1)
Supplement: Supplementary file 1 — Supplementary Information. [file 41598_2021_98560_MOESM1_ESM.pdf]

Detailed characterization of the transcriptome of single B cells in mantle cell lymphoma suggesting a potential use for SOX4

Simone Valentin Hansen[1*], Marcus Høy Hansen[1], Oriane Cédile[1,2], Michael Boe Møller[1], Jacob Haaber[1], Niels Abildgaard[1,3], Charlotte Guldborg Nyvold[1,2,3]

* Corresponding author

1) Hematology-Pathology Research Laboratory, Research Unit for Hematology and Research Unit for Pathology, University of Southern Denmark and Odense University Hospital, Odense, Denmark. 2) OPEN, Odense Patient data Explorative Network, Odense University Hospital, Odense, Denmark. www.sdu.dk/ki/open. 3) CITCO, Centre for Cellular Immune Therapy of Hematological Cancer Odense, Odense University Hospital, Odense, Denmark.

Supplementary material

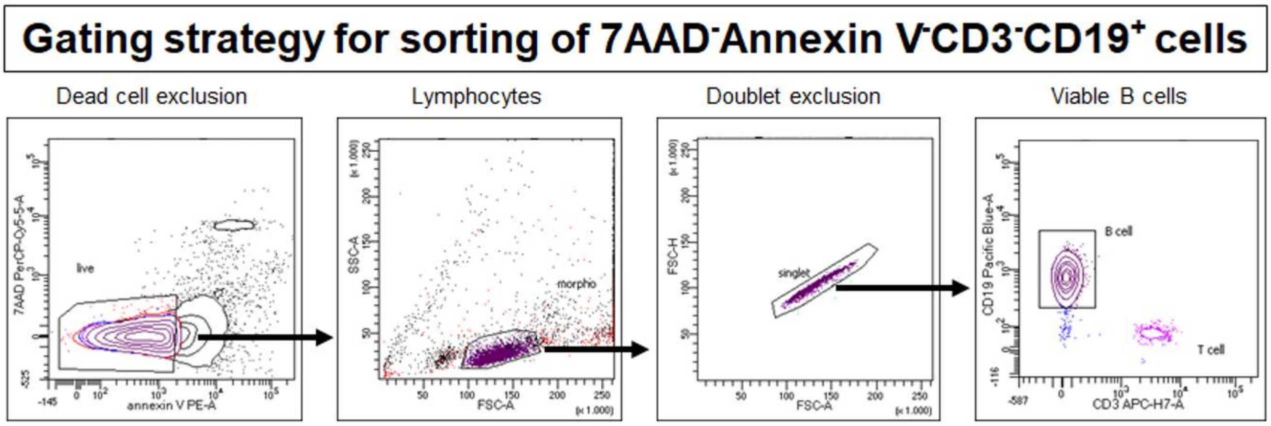

Suppl. Figure 1: Gating strategy for sorting of viable CD19+ cells from MCL bone marrow mononuclear cells. Cryopreserved mononuclear cells from MCL bone marrow were thawed, stained with 7AAD, annexin V, antibodies to target CD19 and CD3 and run on a FACS ARIA III. Viable B cells (7AAD$^-$Annexin V$^-$CD3$^-$CD19$^+$) were sorted and used for single cell RNA sequencing.

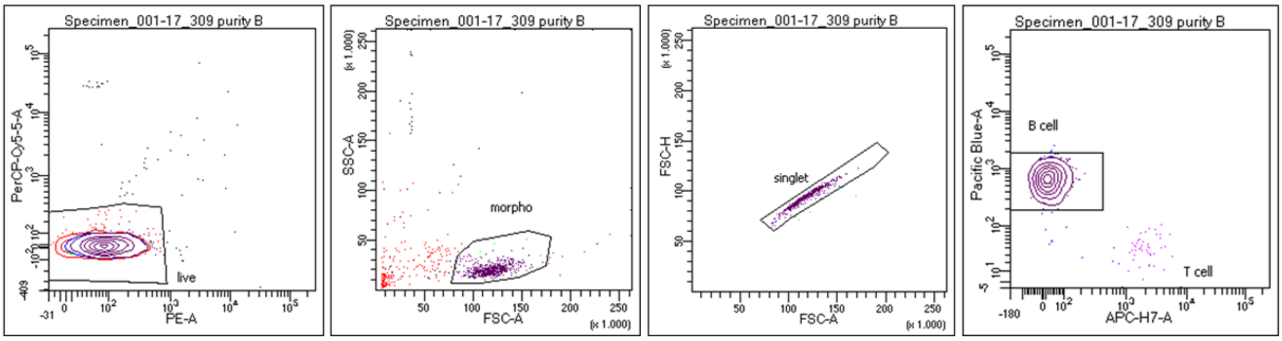

Supplementary Figure 2: Purity of sorted viable B cells (7AAD$^-$Annexin V$^-$CD3$^-$CD19$^+$). An aliquot of each sorted cell population was run again on the FACS ARIA III to check the purity. Representative data is shown in the figure from patient 4, while the purity of all sorted samples is summarized in supplementary Table 1.

Patient/sample	1	2	3	4	5	6	7	8
% from total % from singlet gate	36.6% /99.7%	59.1% /96.8%	13.6% /84.5%	62.8% /90.6%	12.3% /75.6%	58.9% /96.7%	31.6% /97.6%	50% /91.5%

Supplementary Table 1: Purity of sorted 7AAD$^-$Annexin V$^-$CD19$^+$ cells. First % is from total events, second % is from parent gate. The purity of sorted CD19$^+$ cells from total for patient 3 and 5 indicated a higher fraction of apoptotic cells and debris in the latter samples, which was partially accounted for by filtering of dead cells in the data analysis, based on mitochondrial reads (1). For 6 out of 8 patients, the purity from singlets was >90.6%, while being lower for patient 3 (84.5%) and 5 (75.6%), indicating a small contamination with other lymphocytes. However, this is accounted for by positive selection of B cells in the data analysis, based on the assumption that they should express at least one of the chosen B cell markers.

(1) Ilicic T, Kim JK, Kolodziejczyk AA, Bagger FO, McCarthy DJ, Marioni JC, et al. Classification of low quality cells from single-cell RNA-seq data. Genome biology. 2016;17:29.

Patient	1	2	3	4	5	6	7	8
Estimated no. Of cells	3,194	5,082	1,899	6,668	4,081	6,040	1,018	2,583
Mean reads per cell	114,236	82,511	254,028	96,981	25,422	230,884	97,866	33,591
Median genes per cell	505	772	454	862	460	1,151	309	517
Fraction reads in cells	75.5%	82.4%	81,5%	68.9%	81.2%	78.7%	59.3%	76.1%
Total genes detected	19,903	20,101	18,037	21,262	18,690	24,006	15,546	17,629
Median UMI counts pr. Cell	884	2,207	849	2,168	781	2,704	434	871
Reads mapped confidently to genome	71%	88.4%	64.4%	88.7%	92%	93.9%	55.9%	89.2%
Reads mapped confidently to transcriptome	19.1%	49.9%	18.3%	47.2%	14.9%	23.9%	10.8%	13.1%

Supplementary Table 2: Sequencing information from Cell Ranger. For all samples, the percentage of reads confidently mapped to transcriptome was much lower than mapped to genome, indicating many reads in intronic regions. This may be explained by the samples containing a fraction of late apoptotic cells with permeable cell membranes leaking out mRNA leading to a higher fraction of pre-mRNA molecules containing intronic regions as these are contained in the nucleus. Reads confidently mapped to transcriptome was especially low in samples 5, 7 and 8 (supplementary Table 4), suggesting that these samples contained cells with poor cell integrity.

Sample	RIN	RNA conc. (pg/ul)
**1**	NA	65
**2**	8.8	191
**3**	8.8	152
**4**	9.1	379
**5**	NA	139
**6**	8.3	206
**7**	NA	60
**8**	NA	57

Supplementary table 3: RNA integrity number for sorted cells. An aliquot of the sorted fixated cells was used to determine the RNA integrity number (RIN) as a measure of RNA quality. Fixated cells after sorting were rehydrated according to the protocol provided by 10X Genomics, lysed and RNA was extracted using the RNeasy Plus Micro kit (Qiagen). RIN and RNA concentration of each sample were determined using the Bioanalyzer RNA Pico kit (Agilent Technologies) on an Agilent Bioanalyzer 2100. The measurement of RNA quality after cell sorting and methanol fixation showed that patient 4, 2, 3 and 6 had a good RNA quality (RIN> 8.3), while no RIN was determined for patient 1, 7 and 8, potentially due to low RNA concentrations close to the detection limit of the kit. Regrettably, this was not repeated with a higher concentration of RNA, since the sample material was limited. For patient 5, no RIN was determined in spite of this sample having a higher RNA concentration, and the electropherogram showed presence of RNA at lower sizes, suggesting that the RNA of this sample was at least partly degraded.

Patient	1	2	3	4	5	6	7	8	combined
**CCND1**	51.76%	74.63%	17.93%	71.35%	32.88%	69.09%	36.42%	58.68%	60.48%
**CD5**	1.68%	3.81%	1.53%	0.95%	ND	7.14%	1.19%	2.54%	2.89%
**CD10**	1.94%	1.43%	2.32%	0.77%	5.55%	0.11%	4.90%	1.65%	1.69%
**CD19**	11.17%	8.41%	6.11%	22.37%	2.11%	14.01%	6.23%	6.11%	12.24%
**CD20**	76.03%	61.48%	50.13%	91.23%	23.02%	91.57%	46.75%	71.72%	71.25%
**CD23**	1.63%	5.67%	2.46%	8.30%	2.67%	7.37%	2.52%	3.43%	5.62%
**CD27**	0.56%	2.96%	1.13%	8.27%	0.43%	6.65%	ND	ND	4.24%
**CD79A**	12.49%	75.98%	17.40%	84.78%	18.56%	78.87%	7.15%	12.01%	58.03%
**CD79B**	6.37%	13.05%	22.38%	41.25%	7.41%	14.48%	14.04%	8.92%	19.63%
**CD138**	ND	ND	ND	ND	ND	ND	ND	ND	ND
**DNTT**	ND	1.49%	ND	ND	0.46%	ND	ND	ND	0.34%
**IGHA1 (IgA)**	5.41%	23.53%	23.90%	11.22%	24.05%	9.42%	31.13%	10.02%	15.60%
**IGHD (IgD)**	5.00%	3.45%	3.72%	38.68%	4.83%	14.21%	2.78%	1.72%	14.85%
**IGHG1 (IgG)**	1.89%	4.28%	4.12%	13.32%	1.33%	7.09%	1.46%	1.65%	6.39%
**IGHM (IgM)**	70.98%	96.87%	61.75%	87.97%	68.79%	97.09%	53.91%	48.87%	83.31%
**IGKC (IgL κ)**	0.61%	98.06%	46.15%	24.66%	35.88%	98.11%	31.79%	18.26%	55.13%
**IGLC2 (IgL λ)**	28.91%	14.70%	12.42%	99.74%	15.99%	3.16%	24.50%	6.93%	34.72%
**ICKC OR IGLC2**	29.37%	99.05%	52.79%	99.88%	46.14%	98.17%	49.27%	24.02%	79.01%
**IGKC AND IGLC2**	0.15%	13.71%	5.78%	24.52%	5.73%	3.11%	7.02%	1.17%	10.84%
**IGLL1**	ND	2.08%	0.80%	ND	2.45%	ND	ND	0.00%	0.78%
**LDHA**	3.31%	12.56%	6.11%	12.47%	4.18%	15.21%	2.52%	3.57%	10.21%
**LDHB**	1.63%	10.36%	3.85%	19.53%	1.80%	12.55%	ND	0.89%	10.24%
**PAX5**	16.37%	33.64%	32.20%	34.24%	12.71%	42.11%	23.71%	20.86%	30.63%
**SOX4**	14.18%	3.13%	14.08%	1.15%	7.38%	0.38%	16.03%	3.43%	4.42%
**SOX11**	36.97%	36.68%	25.43%	36.37%	12.46%	64.61%	9.80%	25.12%	37.51%
**SOX12**	ND	ND	ND	ND	ND	ND	ND	ND	ND
**SOX11 AND CD23**	0.51%	2.14%	0.60%	3.36%	0.37%	5.25%	0.40%	0.69%	2.55%
**SOX11 AND CCND1**	21.93%	29.88%	6.71%	27.72%	5.55%	47.20%	5.56%	17.30%	26.68%
**SOX11 AND CD19**	4.79%	3.77%	2.26%	9.12%	0.28%	10.07%	0.66%	1.78%	5.82%
**VPREB1**	ND	1.83%	ND	0.15%	1.36%	ND	1.59%	ND	0.60%
**VPREB3**	2.09%	8.43%	2.79%	42.19%	1.03%	14.69%	1.32%	0.82%	16%

Supplementary Table 4: Expression of markers used in lymphoma diagnostics and markers of B cell differentiation identified by scRNA-seq in all cells. The percentage of all cells expressing relevant clinical markers and markers related to B cell differentiation states shown per patient and as the percentage from all patient cells (combined). In concordance to the clinical laboratory results, all patients were positive for SOX11 and CCND1, even though both of these markers was not expressed in all the cells. Generally, the combined population was transcriptionally enriched for $CD20^{+}CD5^{-}CD19^{-}CD23^{-}CD27^{-}$ cells, although the profiles varied patient-wise. While 79.01% of all cells expressed either IGKC or IGLC2, 10.8% of all cells were found positive for both κ and λ Ig light chain genes. 4.42% of all cells expressed SOX4, which is a marker of immature B cells along with DNTT, VPREB1, IGLL1, and CD10 which were all expressed in a very low fraction of cells. A very low percentage of positive cells could be due to background/noise. ND: not detected.

Patient	1	2	3	4	5	6	7	8	combined
CCND1	59.31%	81.44%	26.37%	76.23%	44.53%	73.10%	56.76%	68.85%	71.12%
CD5	2.07%	4.17%	2.87%	1.04%	0.00%	7.57%	2.70%	2.73%	4.21%
CD10	2.07%	0.60%	ND	0.87%	1.24%	0.08%	5.41%	0.27%	0.61%
CD19	12.97%	10.28%	8.88%	25.06%	2.24%	15.59%	6.76%	7.10%	15.53%
CD20	85.52%	67.48%	67.36%	94.10%	32.09%	94.02%	78.38%	83.61%	84.32%
CD23	1.38%	5.84%	2.35%	9.23%	2.99%	8.13%	4.05%	2.73%	6.80%
CD27	0.55%	2.98%	0.26%	9.64%	0.50%	7.89%	ND	ND	5.90%
CD79A	11.31%	78.95%	21.93%	87.57%	21.14%	81.66%	12.16%	11.75%	69.44%
CD79B	8.14%	13.20%	26.89%	46.59%	2.24%	16.34%	8.11%	11.20%	22.15%
CD138	ND	ND	ND	ND	ND	ND	ND	ND	ND
DNTT	ND	0.76%	ND	ND	ND	ND	ND	ND	0.14%
IGHA1 (IgA)	6.07%	24.03%	25.07%	11.43%	30.10%	9.49%	35.14%	10.93%	14.16%
IGHD (IgD)	6.62%	3.30%	5.74%	43.14%	5.22%	15.28%	4.05%	0.82%	17.81%
IGHG1 (IgG)	3.59%	4.27%	2.09%	14.63%	1.00%	7.40%	1.35%	0.82%	7.54%
IGHM (IgM)	72.00%	98.65%	70.23%	91.35%	75.87%	97.72%	62.16%	51.64%	90.47%
IGKC (IgL κ)	0.41%	99.62%	59.79%	25.81%	41.54%	98.52%	48.65%	18.85%	66.41%
IGLC2 (IgL λ)	34.34%	14.18%	11.49%	99.79%	13.93%	3.34%	21.62%	7.65%	32.42%
ICKC OR IGLC2	34.48%	99.68%	63.19%	99.79%	50.00%	98.58%	59.46%	25.41%	87.94%
IGKC AND IGLC2	0.28%	14.12%	8.09%	25.81%	5.47%	3.28%	10.81%	1.09%	10.89%
IGLL1	ND	1.41%	0.26%	ND	0.25%	ND	ND	ND	0.29%
LDHA	4.69%	14.72%	7.05%	14.13%	5.22%	16.25%	4.05%	5.74%	13.29%
LDHB	1.79%	11.96%	4.18%	22.90%	1.00%	13.33%	ND	1.64%	13.17%
PAX5	21.24%	37.82%	39.43%	37.49%	12.69%	45.23%	32.43%	24.32%	37.72%
SOX4	19.68%	1,19%	13.32%	1.21%	1.24%	0.31%	12.16%	0.82%	2.80%
SOX11	100.00%	100.00%	100.00%	100.00%	100.00%	100.00%	100.00%	100.00%	100.00%
SOX12	ND	ND	ND	ND	ND	ND	ND	ND	ND
VPREB1	ND	0.97%	ND	0.08%	0.75%	ND	ND	ND	0.23%
VPREB3	2.90%	7.41%	3.13%	45.72%	0.25%	16.42%	ND	0.55%	19.02%

Supplementary Table 5: Expression of markers used in lymphoma diagnostics and markers of B cell differentiation identified by scRNA-seq in SOX11$^+$ cells. The percentage of SOX11$^+$ cells expressing relevant clinical markers and markers related to B cell differentiation states shown per patient and as the percentage from all cells (combined). Generally, the combined population could transcriptionally be defined as CD20$^+$CD5$^-$CD19$^-$CD23$^-$CD27$^-$ cells with pt. 5 almost completely devoid of measurable CD5 and CD19 in the SOX11$^+$ population. Three patients (1, 3 and 7) harbored a substantial SOX4 positive fraction within the SOX11 expressing cells of 19.68%, 13.32% and 12.16%, respectively. 10.89% of SOX11$^+$ cells were found positive for both κ and λ Ig light chain genes, although being overall consistent with the clinical flow cytometry analysis. While 90.47% of all SOX11$^+$ cells were positive for IgM (range: 51.64%–98.65%), only 17.81% expressed IgD (range: 0.82%–43.14%). All patients harbored SOX11$^+$ cells expressing IgA (range: 6.07%–43.14%), IgG (range: 0.82%–14.63%), and a small SOX11$^+$CD27$^+$ fraction was detected in patient 2, 4 and 6. Additionally, minor compartments of SOX11$^+$CD23$^+$ cells were detected in all patients (range: 1.38–9.23%). A very low percentage of positive cells could be due to background/noise. ND: not detected.

Supplementary methods

Fixation and rehydration of sorted cells for single cell RNA sequencing and for RNA quality check

All sorted 7AAD$^-$Annexin V$^-$CD3$^-$CD19$^+$ B cells were fixated with a mixture of 4/5 100% methanol (Sigma-Aldrich, St. Louis, Mi, USA) and 1/5 RNasin Ribonuclease inhibitor (Promega, Madison, WI, USA), according to the protocol provided by 10x Genomics, and stored at -80 °C until use. Each

sample was examined by microscopy using trypan blue (Invitrogen, Carlsbad, CA, USA) to check for fixation efficiency, debris and ensuring that the cells appeared as single cells. Before using the samples for single cell RNA sequencing, fixated cells were rehydrated in a mixture of 0.04% ultrapure BSA (Invitrogen, Carlsbad, CA, USA), 1mM ultrapure DTT (Invitrogen, Carlsbad, CA, USA), 0.2 U/µl RNasin Ribonuclease inhibitor (Promega, Madison, WI, USA), 3x saline-sodium citrate buffer (SSC buffer; Invitrogen, Carlsbad, CA, USA) according to the protocol from 10X Genomics. As a control of the RNA quality, the Bioanalyzer RNA 6000 Pico Kit (Agilent Technologies, CA, USA) was used to measure the RNA integrity number (RIN) for each sample.
